# Effects of Nut Intake on Gut Microbiome Composition and Gut Function in Adults: A Systematic Review and Meta-analysis

**DOI:** 10.1016/j.advnut.2025.100465

**Published:** 2025-06-13

**Authors:** Matthew Snelson, Jessica R Biesiekierski, Susanna Chen, Nessmah Sultan, Barbara R Cardoso

**Affiliations:** 1 Hypertension Research Laboratory, School of Biological Sciences, Faculty of Science, Monash University, Victoria, Australia; 2 Victorian Heart Institute, Monash University, Clayton, Victoria, Australia; 3 Department of Nutrition, Dietetics and Food, Monash University, Victoria, Australia; 4 Human Nutrition Group, School of Agriculture, Food and Ecosystem Sciences, The University of Melbourne, Victoria, Australia; 5 Department of Neuroscience, School of Translational Medicine, Monash University, The Alfred Centre, Victoria, Australia

**Keywords:** nuts, diet, gut, microbiome, microbiota, short-chain fatty acids

## Abstract

The reduced risk of chronic diseases such as cardiovascular disease and type 2 diabetes associated with nut consumption may occur via modulation of the gut microbiota, although this has not been comprehensively assessed. This systematic review of clinical trials aimed to assess the effects of nuts on gut microbiota composition and metabolites, as well astheir effects on gut function and symptoms in adults. The systematic review was conducted following PRISMA guidelines and registered in PROSPERO (CRD42023451282). Outcomes included microbiota diversity, specific bacterial abundances, gastrointestinal symptoms, intestinal permeability, fecal pH, fecal moisture, and short-chain fatty acid (SCFA) concentrations. We performed meta-analyses to assess the overall effect of nuts on fecal moisture, pH, intestinal permeability, and SCFA concentrations. Among the 28 intervention trials included in this review, almonds were the most commonly studied (12 trials), whereas other nuts, such as walnuts, peanuts, pistachios, and Brazil nuts, were also examined. Nineteen articles reported the effects of almond, walnut, peanut, or mixed nuts on the microbiota composition. Additionally, 6 trials used interventions involving a mixture of different nuts. A total of 19 trials assessed the community structure of the gut microbiota by evaluating α-diversity and β-diversity metrics, with most finding no significant differences following the nut intervention. Regarding taxonomic changes, the majority of studies reported no significant changes across nut interventions. However, several studies noted increases in *Clostridium* and *Roseburia* species, with mixed results for *Bifidobacterium* species abundance following almond or walnut intervention. Five studies assessed fecal SCFA concentrations, with positive effects of nut interventions on propionate. There were no effects of nut interventions on fecal pH and intestinal permeability, with an unfavorable effect on fecal moisture. In summary, the available evidence indicates that nuts have modest effect on gut health, but the substantial heterogeneity between studies may hinder further conclusions.

This trial was registered at PROSPERO as CRD42023451282.

**Statement of significance**

Nuts, recognized for their health benefits, nutrient density, and potential positive effects on the gut microbiota, represent a valuable dietary component. In this study, we examined their impact on gut health outcomes, including microbial diversity and metabolites, short-chain fatty acid production, and gastrointestinal symptoms through a comprehensive analysis of intervention trials.

## Introduction

Nuts are known for their rich nutritional profile, with a high concentration of unsaturated fatty acids and low concentration of saturated fatty acids, in addition of high concentration of vitamins, minerals, proteins, and fibers, as well bioactive compounds such as polyphenols [1,2]. Nuts are a key aspect of healthy dietary patterns such as the Mediterranean diet and the Dietary Approaches to Stop Hypertension diet [3,4] and, more recently, have gained even more attention as an alternative plant-based source of proteins in sustainable diets [5]. Studies have shown that the inclusion of nuts in the diet brings important benefits to human health, with strong evidence associating the regular consumption of nuts with a reduced risk of diabetes [[6], [7], [8]], cognitive impairment [9], and cardiovascular diseases [10,11]. More recently, an umbrella review reported that a daily serving of nuts (28 g) was associated with 21% risk reduction of cardiovascular disease, 11% reduction of cancer-related deaths, and 22% reduction in all-cause mortality [12].

The specific metabolic pathways through which nuts confer their health benefits have yet to be fully understood. The particular nutritional composition of nuts has led researchers to investigate the prebiotic effects of nuts, with experimental research demonstrating that nuts, such as almonds, are capable of increasing the proliferation of beneficial bacteria like *Bifidobacterium* and *Lactobacillus* species *in vitro* [[13], [14], [15]]. Nuts contain substantial amounts of fiber and polyphenols, which can serve as substrates for bacterial fermentation in the colon. The fiber components undergo microbial fermentation to produce short-chain fatty acids (SCFAs), particularly butyrate, which provide energy for colonocytes and help maintain gut barrier function [16]. Additionally, the intact cell walls of nuts may act as a physical barrier, allowing undigested nutrients like lipids to reach the colon where they can be metabolized by the gut microbiota [17]. Through these mechanisms, regular nut consumption may beneficially modulate the gut microbiome composition and activity, potentially contributing to their observed health benefits.

Findings from experimental studies have fostered research in humans to investigate the effects of nuts in gut health, with particular emphasis on the gut microbiome. This systematic review of clinical trials aims to *1*) present up-to-date evidence regarding the effects of nuts on gut microbiota composition and metabolites in adults and *2*) examine the effects of nuts on gut function and symptoms in this population. For the purpose of this review, we considered the following tree nuts: almond, hazelnut, macadamia, pistachio, walnut, pecan, pine nut, Brazil nut, and cashew. Additionally, we considered peanuts, which present with a similar nutritional profile as tree nuts despite being botanically classified as legumes [18].

## Methods

### Study identification and eligibility

This review was conducted in accordance with the PRISMA statement and was prospectively registered in the International PROSPERO (CRD42023451282). The systematic search was carried out in Ovid MEDLINE, Cochrane Central Register of Controlled Trials (CENTRAL), CINAHL, and EMBASE from inception to 21 December, 2023, and repeated on 7 July, 2024. An additional search was conducted on Google Scholar, which was limited to the first 400 results. The acronym PICOS was used to formulate the research questions as follows—*1*) population: adults (≥18 y); *2*) intervention: treatment for ≥1 wk with ≥1 type of whole nut (almond, hazelnut, macadamia, pistachio, walnut, pecan, pine nut, Brazil nut, cashew, and peanut), which could be processed in different ways (e.g., chopped, ground, nut butter, and with/without skin); *3*) comparison: placebo intervention or nut-free diet; *4*) outcomes: primary—gut microbiota assessed as fecal global microbiota composition, α-diversity or β-diversity, concentration of the following species: *Bifidobacterium* spp, *Lactobacillus* spp, *Roseburia* spp, *Akkermansia municiphila*, *Eubacterium hallii*, *Eubacterium rectale*, *Faecalibacterium prausnitzii*, and *Ruminococcus bromii*; secondary—fecal or plasma SCFAs; gastrointestinal symptoms; stool frequency and consistency; differences in bacterial taxa not previously specified as primary outcomes; fecal, urine, and serum/plasma metabolites generated by the gut microbiota; *5*) study design: randomized and nonrandomized trials, with parallel or crossover design. In the case of crossover trials, only trials with a washout period were included. The 4 electronic databases were searched using the following terms: [Nut OR nut consumption OR (juglans OR walnut∗) OR (anacardium OR cashew∗) OR (prunus dulcis OR almond∗) OR (corylus OR hazelnut∗) OR (pistacia or pistachio∗) OR (carya OR pecan∗) OR (pinus OR pine nut∗) OR (bertholletia or brazil nut∗)] AND [(microbiome/ OR microbiome) OR microbiota OR microflora OR microflora/ OR bifido∗ OR bifidobacterium/ OR lactobacill∗ OR lactobacillus/ OR (faecal OR fecal) OR bacteri∗ OR bacterium/ OR (colon flora/ OR bacterial flora/ OR intestine flora/ OR flora/) OR (colon or bacteri∗ OR intestine∗ adj2 flora) OR dysbiosis OR dysbacteriosis]. The search on Google Scholar used the following terms: “nuts microbiome|dysbiosis clinical trials.” The search strategy is presented in Supplemental Table 1.

Eligible studies were published in English, Portuguese, or Spanish. Letters, reviews, conference abstracts, personal opinion articles, case reports, and observational studies were excluded. Further, studies that combined nuts with other interventions that could have precluded the assessment of the effect of the nut consumption alone, did not assess the effect of whole nuts (e.g., nut oils or nut extracts), or did not assess any outcome of interest were also excluded.

### Screening and data extraction

All references resulting from the search were transferred to EndNote 21 (Clarivate), where duplicates were identified and removed. The references were subsequently uploaded into Covidence (Covidence Systematic Review Software; Veritas Health Innovation), a systematic review screening and data extraction software program. This software was used to screen studies according to the eligibility criteria. As the first step, the Covidence “Auto-marked as ineligible” feature was used to automatically remove noncontrolled trial articles. The remaining articles underwent a 2-phase screening process by 2 of the listed authors (SC, BRC, JRB): in the first phase, the authors screened article titles and abstracts; in the second phase, the selected articles underwent full-text screening. Conflicts were collaboratively resolved among the 3 authors (BRC, JRB, MS). The PRISMA flowchart was automatically generated by the Covidence program after the screening completion (Supplemental Figure 1).

A single author (BRC or SC) conducted the data extraction, subsequently verified by a second author (BRC or JRB) on Google Sheets. Data collected included first author, year of publication, study characteristics (design, country, and inclusion/exclusion criteria), study population (gender, age, and BMI), intervention details (type and quantity of nut, presentation, processing, length, run in and washout periods, comparator, and compliance), and primary and secondary outcomes. When outcomes of interest were collected but remained unreported, the corresponding authors were contacted by email to obtain the necessary information. Where data were available only in graphical format, a validated freely available online program, WebPlot digitizer (version 4.7), was used to extract data [19]. Where mean and the SD were not available, we instead collected the median and first and third quartiles and/or minimum and maximum values and estimated the mean and SD with the *estmeansd* package (version 1.0.1) using the Box-Cox method, which does not rely on the assumption of normality [20].

### Risk of bias

Risk of bias (RoB) was conducted using Cochrane RoB2 for randomized trials [21] or risk of bias in nonrandomized studies – of interventions (ROBINS-I) tool [22] by 2 of the listed authors (NS, SC, JRB). The RoB2 tool detects potential biases by evaluating 5 aspects: randomization bias, deviations from planned interventions, incomplete data, bias in outcome measurement, and selective reporting of results. RoB for each study was determined as low risk, some concerns, or high risk. The ROBINS-I tool assesses potential biases in nonrandomized studies across 7 domains: confounding, participant selection, intervention classification, deviations from intended interventions, incomplete outcome data, outcome measurement bias, and selective reporting. Each study’s RoB was categorized as follows: no information, low risk, moderate risk, serious risk, or critical risk. Inconsistencies between the authors’ RoB assessments were resolved through collaborative discussion until a consensus was reached.

### Data analysis

Owing to substantial methodological heterogeneity across trials in microbiota analysis—from sample collection through to bioinformatic processing and reporting—statistical pooling of microbiota data was not conducted as it would not yield valid effect estimates. Even commonly reported metrics like α-diversity were challenging to compare between studies due to variations in sequencing depth and analytical approaches [23]. Therefore, the microbiota findings are presented narratively.

A meta-analysis assessing the overall effect of intervention with any nut type was carried out for fecal pH, fecal moisture, intestinal permeability, and SCFAs (acetate, propionate and butyrate concentrations) using Review Manager (RevMan; version 8.17; The Cochrane Collaboration 2024). Crossover trials were excluded from the meta-analysis due to concerns about carryover effects and challenges in appropriately accounting for the within-subject correlation in the analysis [24]. The standardized mean difference (SMD) was determined with 95% CIs from endpoint values as the effect measure to account for variations in outcome measurements across studies. Sensitivity analyses were conducted by omitting studies that used nut butter or nut powder interventions, as opposed to whole nut interventions, to assess the influence of processing on outcomes. A network meta-analysis was initially planned as per our PROSPERO registration; however, the high degree of heterogeneity between studies hindered such analysis.

## Results

### Study selection

The first database search retrieved 9517 records. After excluding duplicates (*n* = 7483), 2034 records remained for the screening process. After screening titles and abstracts, 1982 records did not meet the eligibility criteria and were removed. In the search update, 309 studies were screened and 8 had titles and abstracts assessed for eligibility. From these, only 2 were included in the systematic review. In total, 37 articles were full-text assessed for eligibility, and 28 met the eligibility criteria and were included in this systematic review (Supplemental Figure 1).

### Study characteristics

The 28 articles included in this systematic review, published between 2012 and 2024, represent 23 different trials involving 1324 participants with data for the outcomes of interest. A total of 12 articles presented data from crossover studies [[25], [26], [27], [28], [29], [30], [31], [32], [33], [34], [35], [36]], whereas 16 represented parallel trials—13 with 2 arms [[37], [38], [39], [40], [41], [42], [43], [44], [45], [46], [47], [48], [49], [50]] and 2 with 3 arms [51,52]. Two of the included trials were not randomly assigned [31,43]. The articles reported the effects of nuts in healthy adults [[25], [26], [27],^29^,^30^,^36^,^38^,^39^,^42^,^43^,^49^,^51^,^52^], people with high cardiovascular disease risk [32,33,35,40,41,[44], [45], [46],^50^] and diabetes or increased risk of it [28,34,37,48], healthy people with obesity [47], and patients on hemodialysis [31]. Only 6 studies excluded participants who were habitual nut consumers [28,41,42,48,50,51]. The duration of intervention in the trials varied across the studies: the shortest trial was conducted for 18 d [36], with 4 interventions conducted for 3 wk [26,29,30,49], 4 interventions conducted for 4 wk [27,31,32,51], 5 articles reporting on interventions that lasted for 6 wk [[33], [34], [35],^40^,^43^], 6 reporting on 8-wk trials [25,[37], [38], [39],^41^,^50^], 7 reporting on trials that were conducted from 12 wk to 4 mo [28,42,[44], [45], [46], [47], [48]], and the longest trial conducted for 6 mo [52]. Fourteen studies had a run-in period, which ranged from 6 to 10 d [26,46,[48], [49], [50]] and 2 [28,31,32,33,35,37,40,43,52] or 4 wk [25]. The crossover trials had an overall washout period ranging from 1 to 6 wk [[25], [26], [27], [28], [29], [30], [31], [32], [33], [34], [35], [36]]. The control group of most studies either received energy-matched foods (crackers, muffins, potato chips, and rice bars; *n* = 10) or followed nut-free diets with no provision of specific foods (*n* = 15). Microbiota data were reported in 20 articles [[25], [26], [27],[29], [30], [31],[33], [34], [35], [36], [37], [38],^41^,^42^,[46], [47], [48], [49], [50], [51]], with all except 2 [33,41] using 16S rRNA sequencing (methodological details of 16S rRNA sequencing in Supplemental Table 2). Ghanavati and Nasrollahzadeh [41] used qPCR to target specific bacterial genera (*Bacteroides*, *Prevotella*, *Bifidobacterium*, and *Lactobacillus*). Six studies reported the fecal concentration of SCFAs [32,37,40,[50], [51], [52]], 4 reported on fecal pH [37,43,50,51], 2 on fecal moisture [37,43], and 3 on other fecal metabolites [30,43,47], whereas 3 studies assessed microbiota-related metabolites in plasma [39,44,47]. Intestinal permeability was assessed in 2 studies [37,50], and 6 articles assessed gastrointestinal symptoms [25,26,31,32,49,51]. Table 1 summarizes the characteristics of the studies included in this systematic review.

**TABLE 1:** Study characteristics.

Reference	Study design	No. of participants; age (y); % female	Type and quantity of nuts consumed	Control intervention	Intervention duration	Run-in	Washout
Bamberger et al., 2018 [25]	Randomized crossover	135 healthy adults; mean age: 63 ± 7 y; 66% female	43 g walnuts (shelled) in addition to one of the following dietary modifications: *1*) reduce fat (30 g of saturated fat), *2*) reduce carbohydrates (70 g), *3*) reduce both fats and carbohydrates (35 g carbohydrates and 15 g fats)	Usual diet	8 wk	4 wk	4 wk
Burns et al., 2015 [26]	Randomized crossover	28 healthy adults; mean age: 35 ± 0.6 y; 83% female	42.5 g almonds (whole nut or butter)	Usual diet	3 wk	1 wk	6 wk
Choo et al., 2020 [37]	Randomized parallel	69 adults with overweight or obesity and elevated fasting blood glucose; mean age: 60.8 ± 6.1 y (almond group), 60.8 ± 8.4 y (control group); 36% female (almond group), 50% female (control group)	56 g of raw almonds (raw)	Energy-matched biscuits	8 wk	2 wk	NA
Creedon et al., 2022 [51]	Randomized parallel (3 arms)	74 healthy adults; mean age: 27.5 ± 6.2 y; 86.2% female	56 g of almonds (raw)	Energy-matched muffin	4 wk	No	NA
Dhillon et al., 2019 [38]	Randomized parallel	73 healthy adults; 100% participants of almond group were 18 y, 97% of control group were 18 y, and 3% were 19 y; 57.9% female (almond group), 54.3% female (control group)	57 g of almonds (dry roasted)	Energy-matched crackers	8 wk	No	NA
Dhillon et al., 2023 [39]	Randomized parallel	73 healthy adults; 100% participants of almond group were 18 y, 97% of control group were 18 y, and 3% were 19 y; 57.9% females (almond group), 54.3% females (control group)	57 g of almonds (dry roasted)	Energy-matched crackers	8 wk	No	NA
Dikariyanto et al., 2020 [40]	Randomized parallel	105 people with CVD risk; mean age: 56.3 ± 10.2 y (almond group), 50.0 ± 10.7 y (control group); 70% females (almond), 71% females (control)	Almonds to provide 20% estimated energy requirement	20% estimated energy requirement muffins	6 wk	2 wk	NA
Ghanavati and Nasrollahzadeh et al., 2023 [41]	Randomized parallel	67 people with coronary artery disease; mean age: 58 ± 7 y (nuts group), 59 ± 8 y (control group); 45.7% females (nuts group), 43.7% females (control group)	Mixed nuts (almond, peanut, and pistachio) to provide 20% estimated energy intake of an energy-restricted diet	Nut-free energy-restricted diet	8 wk	No	NA
Haskell-Ramsay et al., 2023 [27]	Randomized crossover	79 healthy people; mean age: 29.9 ± 8.12 y; 65% female	30 g mixed nuts (15 g walnuts, 7.5 g almonds, 7.5 g hazelnuts)	Placebo (microcrystalline cellulose)	4 wk	No	4 wk
Hernández-Alonso et al., 2017 [28]	Randomized crossover	39 people with prediabetes; mean age: 55.3 (95% CI: 53.2, 57.4) y; 49% female	57 g pistachio (roasted)	Energy-matched control diet	4 mo	15 d	2 wk
Herselman et al., 2022 [42]	Randomized parallel	25 healthy people; mean age: 22 y; 35% female (walnut group), 40% control group	56 g walnuts	Usual diet	16 wk	No	NA
Holscher et al., 2018 [30]	Randomized crossover	18 healthy people; mean age: 56.7 ± 10.2 y; 44% female	42 g almonds (whole natural; whole roasted; chopped roasted; butter, roasted)	Energy-matched typical American diet	3 wk	No	1 wk
Holscher et al., 2018 [29]	Randomized crossover	18 healthy people; mean age: 53.1 (SEM: 2.2) y; 44% female	42 g walnuts	Energy-matched diet	3 wk	No	1 wk
Lambert et al., 2020 [31]	Crossover (nonrandomized)	20 patients on hemodialysis; median age: 67.5 (IQR: 57.5–77.7) y; 49% female	40 g almonds	Usual diet	4 wk	2 wk	2 wk
Liu et al., 2014 [43]	Parallel (nonrandomized)	46 healthy people; age range: 18–22 y; 50% female	10 g almond skin powder	Fructooligosaccharides(8 g/d)	6 wk	2 wk	2 wk
Mora-Cubillos et al., 2015 [44]	Randomized parallel	47 people with metabolic syndrome; mean age: 51.8 (range 26–63) y; 40% female (nut group), 48% female (control group)	30 g mixed nuts (15 g walnuts, 7.5g almonds, 7.5g hazelnuts; raw unpeeled) + recommendations to follow the American Heart Association dietary guidelines	Recommendations to follow the American Heart Association dietary guidelines	12 wk	No	NA
Nishi et al., 2021 [32]	Randomized crossover	22 people with hyperlipidemia; mean age: 64.5 ± 9 y; 45% female	73 ± 5 g almonds or 38 ± 3 g almonds + muffins	Energy-matched muffins	4 wk (1 mo)	2 wk	2 wk
Parilli-Moser et al., 2021 [52]	Randomized parallel (3 arms)	63 healthy people; mean age: 22.28 ± 3.20 y (skin-roasted peanut group), 23.43 ± 2.90 y (peanut butter group), 22.42 ± 3.29 y (control butter)	32 g peanut butter; 25 g skin-roasted peanuts	Energy-matched control butter made with peanut oil, free of polyphenols, and fiber	6 mo	2 wk	NA
Petersen et al., 2023 [33]	Randomized crossover (3 arms)	36 overweight people with CVD risk; mean age: 43 ± 10 y; 40% female	Walnuts to provide 18% estimated energy requirement	Fatty acid-matched diet devoid of walnuts Diet where oleic acid replaces α-linolenic acid	6 wk	2 wk	22.8 d (mean)
Ren et al., 2020 [48]	Randomized parallel	45 people with type 2 diabetes; mean age: 73.55 ± 4.99 (almond group), 70.48 ± 5.91 (control group); 59% female	56 g almonds (replacing 150 g/d of carbohydrate-rich staple food)	Low-fat diet education program	3 mo	1 wk	NA
Rosas et al., 2023 [49]	Randomized parallel	20 healthy people; mean age: 24.4 y; 85% female	42 g of mixed nuts (almonds, Brazil nuts, cashews, macadamia, peanuts, pecans, pistachios, and walnuts)	Energy-matched lightly salted potato chips	3 wk	7 d	NA
Sapp et al., 2022 [34]	Randomized crossover	48 people with elevated fasting glucose; mean age: 42 ± 15 y, 44% female (peanut group); 52% female (control group)	28 g peanuts (dry-roasted unsalted)	Energy-matched lower-fat higher-carbohydrate snack	6 wk	No	Median: 28 d (range: 28–41 d)
Silveira et al., 2024 [50]	Randomized parallel	19 overweight people with CVD risk; mean age: 31.7 ± 1.8 y; 100% female	30 g cashew nuts + 15 g Brazil nuts	Energy-restricted (−500 kcal/d) diet	8 wk	7-10 d	NA
Tindall et al., 2020 [35]	Randomized crossover	38 overweight people with CVD risk; mean age: 43 ± 2 (range: 30–60) y; 45.2% female	Walnuts to provide 18% estimated energy requirement	Walnut-free fatty acid-matched diet	6 wk	2 wk	Mean: 22.8 (range: 1–164) d
Tulipani et al., 2012 [45]	Randomized parallel	42 people with metabolic syndrome; age range: 31–63 y; 44% female	30 g mixed nuts (15 g walnuts, 7.5 g almonds, 7.5 g hazelnuts; raw, unpeeled)	Usual diet	12 wk	No	NA
Ukhanova et al., 2014 [36]	Randomized crossover	32 healthy people; mean age: 56 (range: 32–67) y (almond group), 50 (range: 29–64) y (pistachio group); % female not informed	42 g/d either almonds or pistachios	Usual diet	18 d	No	7 d
Wang et al., 2021 [46]	Randomized parallel	99 people with metabolic syndrome risk; mean age: 46.2 ± 9.9 y (peanut group), 46.2 ± 9.9 y (control group); 69% female (peanut group), 70% female (control group)	56 g peanuts (roasted lightly salted)	Energy-matched rice bars	12 wk	6 d	NA
Yang et al., 2023 [47]	Randomized parallel	95 people with overweight or obesity; mean age: 48.3 ± 14.1 y (nut group), 46.8 ± 10.7 y (control group); 70% female (nut group), 84% female (control group)	42.5 g mixed nuts (almond, cashew, hazelnut, macadamia, pecan, pistachio, and walnut) as part of a hypocaloric diet	Energy-matched pretzel as part of a hypocaloric diet	12 wk on hypocaloric diet + 12 wk on isocaloric diet	No	NA

Abbreviations: CVD, cardiovascular disease; NA, not available.

### Types of nuts

The effects of almonds were assessed in 12 articles. All except 1 study [43] provided almonds in volumes above the usual recommendation of 30 g/d in different countries [[53], [54], [55]]. The forms of almond intervention varied across the studies, with studies using almonds as whole, chopped or butter, as well as almond skin powder. Six studies added other nuts to almonds to provide a mix of nuts: Tulipani et al. [45], Mora-Cubillos et al. [44], and Haskell-Ramsay et al. [27] used 30 g/d of mixed nuts with walnuts (15 g), almonds (7.5 g), and hazelnuts (7.5 g); Ghanavati and Nasrollahzadeh [41] combined almonds with peanuts and pistachios to provide 20% of the total daily energy intake (amounts ranged from 39 to 60 g/d); Yang et al. [47] provided almonds along with cashews, hazelnuts, macadamias, pecans, pistachios, and walnuts (42.5 g/d); and Rosas et al. [49] combined almonds with Brazil nuts, cashews, macadamia, peanuts, pecans, pistachios, and walnuts (42 g/d). Walnuts were investigated in 5 studies [25,29,33,35,42], which offered 42–99 g/d. Interventions with peanuts were investigated in 3 trials using 25–56 g/d of either butter or roasted peanuts. The intervention protocol studied by Hernández-Alonso et al. [28] consisted of 57 g/d of roasted pistachios, and Souza Silveira et al. [50] investigated the effects of a mix with 30 g/d of cashew nuts and 15 g/d Brazil nuts.

### Risk of bias

Most studies were graded as low or some concerns, with 2 graded as high according to the RoB2 and ROBINS-I tools. For the randomized trials, the main methodological limitations included failure to report the randomization process [[25], [26], [27], [28], [29],^36^,^41^,^42^,[45], [46], [47],^49^], and a lack of information regarding blinding and retention rates [34,36,37,40,44,45,50,52]. Two of the crossover studies received high concerns due to inadequate washout periods [29,30]. Five randomized trials were found to have a low RoB [35,38,39,48,51]. One nonrandomized intervention trial [43] was found to have a serious RoB, due to failure to validly and reliably control for confounding variables (Supplemental Figure 2).

### Gut microbiota changes

A summary of all microbiota findings is presented in Table 2.

**TABLE 2:**
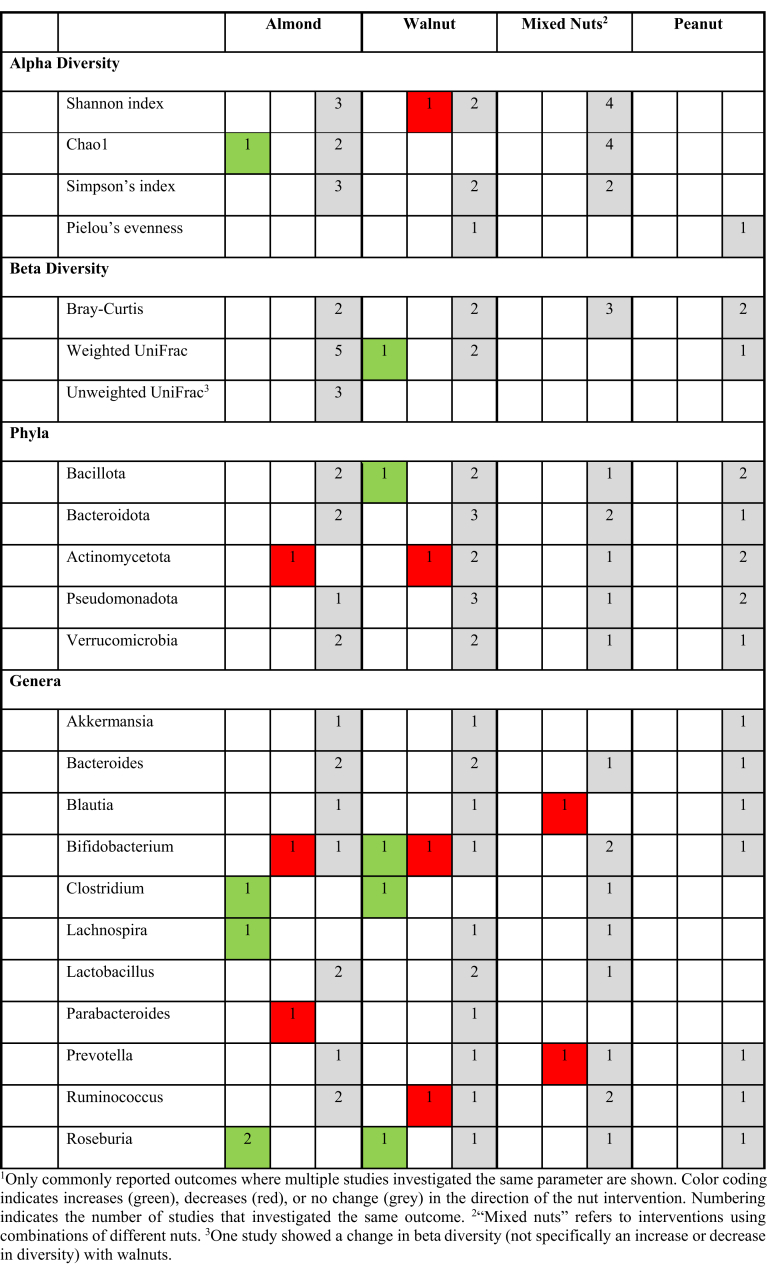
Summary of between-group comparisons in microbiome composition, organized by taxonomic level for the different nut intervention types^1^.

#### Global microbiota composition (α-diversity and β-diversity)

α-Diversity and β-diversity of the microbiota were assessed in 19 trials [[25], [26], [27],[29], [30], [31],[33], [34], [35], [36], [37], [38],^42^,[46], [47], [48], [49], [50], [51]]. The most commonly used α-diversity metric was the Shannon index, evaluated in 10 trials. Only one 16-wk walnut intervention trial demonstrated between-group differences, showing significant decreased α-diversity in the nut intervention group (δ: 0.24) compared with that in the control group (δ: 0.42), specifically among healthy females [42]. The remaining 9 trials (2 walnut, 3 almond, and 4 mixed nut interventions) showed no differences in Shannon index between intervention and control groups [[25], [26], [27],^38^,^46^,^47^,[49], [50], [51]].

Seven trials (3 almond, 2 walnut, and 2 mixed nut interventions) reported no between-group differences in Simpson index [25,26,37,38,[49], [50], [51]]. The Chao1 index was assessed in 7 trials (3 almond and 4 mixed nut interventions), with only 1 almond trial finding a significant increase between the intervention (57 g dry-roasted almond/d for 8 wk) and control groups [38], and the others not [36,[47], [48], [49], [50], [51]]. Two trials (1 with walnuts and 1 with peanuts) evaluated Pielou evenness, both reporting no between-group differences [33,34].

Additional α-diversity metrics including observed species, genes, observed amplicon sequence variants, and Faith phylogenetic diversity were assessed in 12 trials (6 almond, 3 walnut, 2 peanut, and 1 mixed nut intervention). None of these trials demonstrated significant between-group differences [26,27,[29], [30], [31],[33], [34], [35],^37^,^38^,^46^,^48^].

β-Diversity was evaluated using several metrics across trials. Nine trials assessed Bray–Curtis dissimilarity (2 almond, 2 walnut, 3 mixed nut, and 2 peanut trials) [27,33,34,38,42,46,47,49,51], showing no significant between-group differences. Nine trials used weighted UniFrac [25,29,30,34,35,37,38,48,51], where only 1 found significant differences reporting an increase after consuming 3 wk of 42 g walnut/d in 18 healthy adults [29]. Four trials used unweighted UniFrac distances, with 3 reporting no differences [30,38,51] and 1 reporting significant dissimilarities (*P* = 0.02 and *P* = 0.026) after walnut intervention, involving 43 g walnut/d for 8 wk [25].

#### Taxonomic changes—abundances of phylum-level taxa

The effects of nut consumption on bacterial phyla were reported in 8 studies. Largely, there were no significant between-group differences observed. Among studies examining Bacillota (previously called Firmicutes) abundance (3 walnut, 2 almond, 2 peanut, and 2 mixed nut interventions), all but 1 reported no between-group differences [25,[29], [30],^34^,^42^,^46^,[48], [50]]. The exception was a 3-wk trial with 42 g/d walnut consumption that reported an increase in Bacillota abundance [29]. Seven trials measured Actinomycetota (previously called Actinobacteria), with 5 reporting no differences [25,34,42,46,50] and 2 trials reporting a decrease with either almond [30] or walnut intervention [29].

Eight trials (2 with almonds, 3 walnuts, 2 mixed nuts, and 1 peanuts) measured Bacteroidota (previously called Bacteroidetes) abundance, all reporting no between-group differences compared with control interventions [25,27,29,30,34,42,48,50]. Similarly, 7 trials found no differences in Pseudomonadota (previously called Proteobacteria) abundance (3 with walnuts, 1 almonds, 2 peanuts, and 1 mixed nuts) [25,29,30,34,42,46,50], and 6 trials reported no differences in Verrucomicrobia (2 with walnuts, 2 peanuts, 1 almonds, and 1 mixed nuts) [29,30,34,42,46,50].

Several other phyla were investigated in individual studies with no significant differences between nut and control groups: Mycoplasmatota (previously called Tenericutes), Euryarchaeota, Cyanobacteria [34], Lentisphaerae [34], and Fusobacteria [46]. Additionally, 4 studies reported no significant differences in the Bacillota:Bacteroidota (previously called the Firmicutes:Bacteroidetes) ratio after nut intervention [29,41,49,50].

#### Taxonomic changes—abundances of genus-level taxa

At the genus-level, several key taxa showed varying responses to nut consumption. Among genera in the Bacillota phyla, *Blautia* was assessed in 4 studies, with 2 reporting no differences with walnut or almond interventions [29,30], 1 trial involving daily 43 g walnut intervention showing a decrease [25], and another mixed nut showing no difference [50]. Of 7 studies assessing *Ruminococcus*, 6 reported no differences across various nut interventions [27,30,42,46,48,50], whereas 1 walnut intervention showed a decrease [29].

*Roseburia* showed more variable responses across 7 studies: 3 trials (2 with almonds and 1 walnuts) reported increases [29,30,48], whereas 3 trials (1 with peanuts, 1 walnuts, and 1 mixed nuts) found no differences [27,35,46]. *Lachnospira* was examined in 3 studies, with 1 showing increases following almond consumption [30] and 2 showing no difference [27,29] (walnut and mixed nut intervention, respectively).

Among genera in the Bacteroidota phyla, *Bacteroides* was measured in 7 studies, all showing no differences [29,30,41,42,46,48]. *Parabacteroides* showed mixed results in 2 studies, with a decrease following almond intervention [30] but no difference after walnut intervention [29]. *Prevotella* was measured in 5 studies, with 4 showing no difference [30,41,42,46] and 1 mixed nut trial showing a significant between-group decrease [49].

Notably, mixed responses across 8 studies were seen for *Bifidobacterium*: 1 walnut trial reported an increase [25], 2 showed a decrease (almond and walnut interventions) [29,30], and 5 demonstrated no differences [26,36,41,42,46]. Five trials assessed *Lactobacillus*, all showing no differences after nut intervention [29,[41], [42], [43],^48^]. *Clostridium* was assessed in 3 studies, with one 42-g/d almond intervention for 3 wk [30] and one 42-g/d walnut intervention for 3 wk [29] showing an increase, whereas 1 mixed nuts trial reported no differences [27]. Other genera, including *Collinsella* [29,30] and *Akkermansia* [29,30,46], consistently showed no differences between nut and control groups.

#### Taxonomic changes—abundances of species-level taxa

Species-level analyses were less commonly reported across studies. *Bacteroides fragilis* was examined in 1 study, showing a decrease with almond intervention [38]. Two *Prevotella* species (*Prevotella bivia* and *Prevotella disiens*) were measured in 1 trial, both showing decreases after a mixed nut intervention [49]. *Bifidobacterium longum* showed an increase in that same study, which involved administering a mixed nut (almonds, Brazil nuts, cashews, macadamias, peanuts, pecans, pistachios, and walnuts) intervention over 3 wk in 20 healthy adults [49].

#### Taxonomic changes—Archaea composition

Archaea composition was measured in 2 studies investigating almond and walnut interventions [29,30], respectively, with neither finding significant differences compared with control groups.

### Gastrointestinal symptoms and measures

#### Constipation

One parallel [49] and 2 crossover [26,31] studies reported on constipation outcomes. The 2 crossover studies used an almond intervention [26,31], whereas 1 parallel used a mix of nuts containing cashews, almonds, Brazil nuts, pecans, macadamia nuts, peanuts, walnuts, and pistachios [49]. The study on a dialysis population observed reductions in constipation with almond consumption, and Burns et al. [26] noted a trend toward reduced constipation in the final week of the almond intervention. No changes in constipation were observed in the mixed nut study [49].

#### Pain

One parallel [49] and 2 crossover [26,32] studies assessed pain during bowel movements. The interventions included a mixed nut group (almonds, Brazil nuts, cashews, macadamia nuts, peanuts, pecans, walnuts, and pistachios) and almond-specific treatments (half-dose and full-dose). No studies reported any differences in pain with nut intervention [26,32,49].

#### Other gastrointestinal symptoms

Only 1 study reported results for diarrhea and reflux following a nut intervention [26]. Because only a single study was available, a meta-analysis could not be conducted for these outcomes. This study assessed gastrointestinal symptoms using the Gastrointestinal Symptom Response Scale and observed that participants receiving the almond intervention reported less reflux and diarrhea, compared with control [26].

#### Fecal pH

**FIGURE 1:**
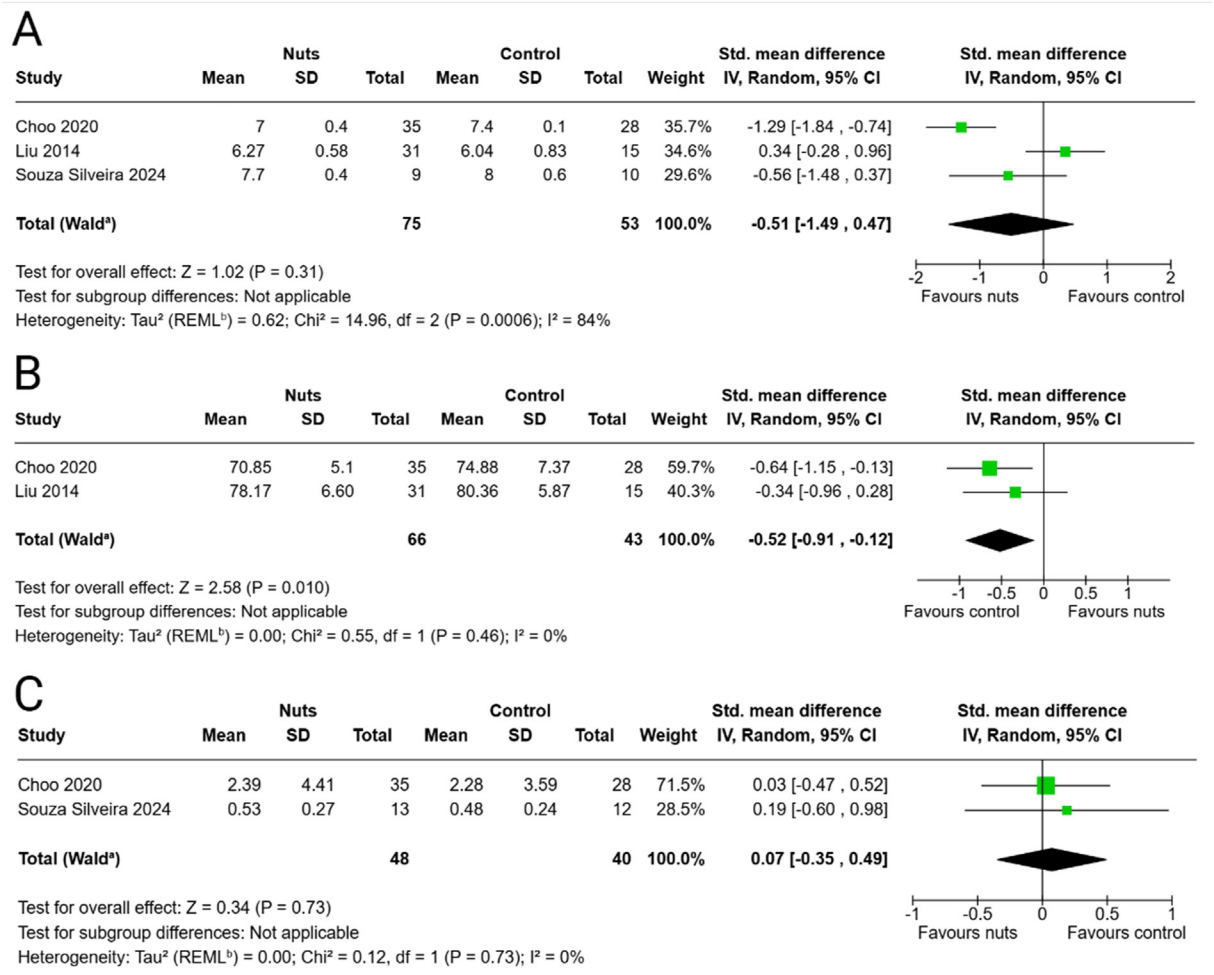
Effects of nut consumption on (A) fecal pH, (B) fecal moisture, and (C) intestinal permeability. Standardized mean difference (SMD) and 95% CI shown for individual and pooled trials. ^a^CI calculated by Wald-type method; ^b^Tau^2^ calculated by restricted maximum-likelihood method.

Three studies reported on fecal pH outcomes following interventions with Brazil nuts, whole almonds, or almond skin powder [37,43,50]. The meta-analysis showed no effects of the nut interventions on fecal pH (SMD: −0.51; 95% CI: −1.49, 0.47) (Figure 1A), and similar findings were observed in the sensitivity analysis without the almond skin powder intervention (Supplemental Figure 3A).

#### Fecal moisture

Two studies examined fecal moisture outcomes [37,43], with the meta-analysis showing that the interventions favored the controls (SMD: −0.52; 95% CI: −0.91, −0.12) (Figure 1B). The sensitivity analysis without the almond skin powder intervention did not change the results (Supplemental Figure 3B).

#### Intestinal permeability

Two studies evaluated the effects of Brazil nuts and almonds on intestinal permeability [37,50]. The meta-analysis revealed no effects of the nut treatments on gut permeability (SMD: 0.07; 95% CI: −0.35, 0.49) (Figure 1C).

### Short-chain fatty acids

Five studies investigated the impact of various nut interventions on fecal SCFAs concentrations [37,40,[50], [51], [52]]. The treatments analyzed included peanuts, whole almonds, ground almonds, peanut butter, and Brazil nuts.

#### Acetate

**FIGURE 2:**
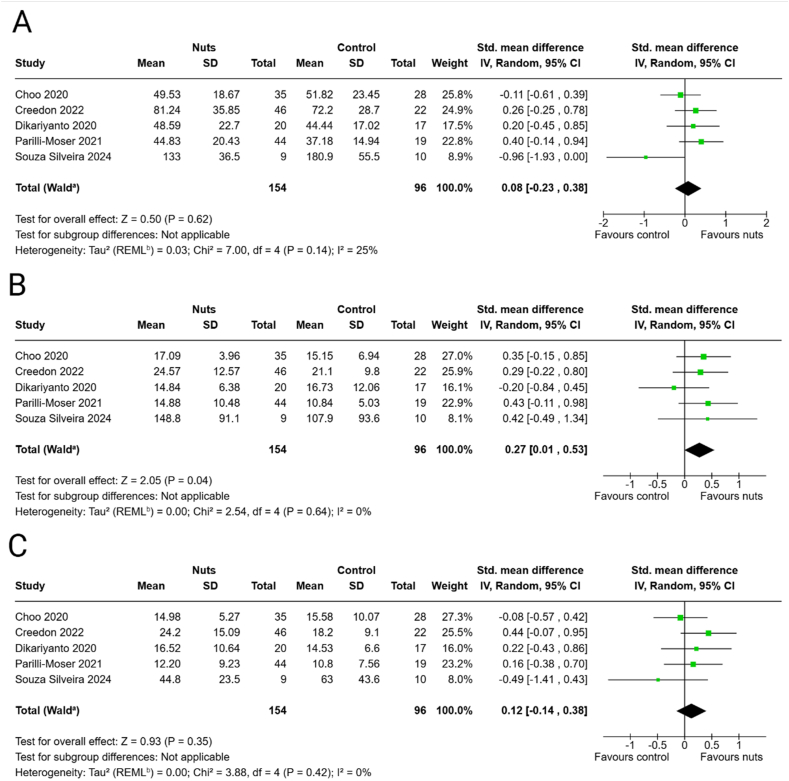
Effects of nut consumption on (A) acetate, (B) propionate, and (C) butyrate concentrations. Standardized mean difference (SMD) and 95% CI shown for individual and pooled trials. ^a^CI calculated by Wald-type method; ^b^Tau^2^ calculated by restricted maximum-likelihood method.

The meta-analysis showed no effects of the nut interventions on acetate concentrations (SMD: 0.08; 95% CI: −0.23, 0.38) (Figure 2A), with similar findings in the sensitivity analysis excluding peanut butter (Supplemental Figure 4A).

#### Propionate

A meta-analysis revealed that the nut interventions resulted in higher concentrations of propionate (SMD: 0.27; 95% CI: 0.01, 0.53) (Figure 2B), with similar findings in the sensitivity analysis without peanut butter (Supplemental Figure 4B).

#### Butyrate

The meta-analysis revealed no effects of the nut interventions on butyrate concentrations (SMD: 0.12; 95% CI: −0.14, 0.38) (Figure 2C); the sensitivity analysis excluding peanut butter did not change the results (Supplemental Figure 4C).

### Plasma gut microbiota–related metabolites

The consumption of 30 g of mixed nuts containing walnuts (15 g), almonds (7.5 g), and hazelnuts (7.5 g) for 12 wk resulted in a higher concentration of urolithin A glucuronide in the plasma of adults with metabolic syndrome than that in the control group [44]. In the study by Yang et al. [47], the intake of a mix of nuts containing almonds, cashews, hazelnuts, macadamias, pecans, pistachios, and walnuts (42.5 g) as part of a hypocaloric diet did not change the plasma concentration of cardioprotective tryptophan microbial metabolites (namely tryotophan, kynurenine, kynurenic acid, indole-3-propionatic acid, indole acetic acid, serotonin, and indole sulphates) in comparison with the control group. Skin-roasted peanuts led to a lower concentration of circulating very long-chain saturated fatty acids (more specifically, arachidic acid and lignoceric acid) than a control peanut oil-based butter (free of phenolic compounds and fibers), whereas peanut butter intake resulted only in a lower concentration of lignoceric acid than control [52]. Dhillon et al. [39] showed that the consumption of almonds (57 g) for 8 wk yielded a higher plasma concentration of phenylacetylglutamine, a conjugate of glutamine and phenylacetate derived from microbial metabolism.

### Urinary gut microbiota–related metabolites

Hernández-Alonso et al. [28] demonstrated that daily consumption of 57 g/d pistachios for 12 wk by people with prediabetes resulted in lower urinary concentrations of hippurate, *p*-cresol sulphate, and dimethylamine than the control group. Tulipani et al. [45] found that the consumption of 30 g/d of a mix containing almonds, hazelnuts, and walnuts increased the excretion of 2 gut microbial-derived phenolic metabolites of ellagitannins (urolithins A and B), whereas no difference was observed for aglycone ellagic acid, flavan-3-ol monomers and oligomers, and proanthocyanidins.

## Discussion

This systematic review presents the most recent evidence of the effects of nuts on gut microbiota composition, gut function, and gastrointestinal symptoms. Our findings revealed predominantly modest and highly variable effects on the gut microbiota composition, these were often nut-specific effects rather than universal across nut types. Our meta-analyses showed no effects of nut interventions on gastrointestinal symptoms, with unfavorable outcomes on fecal moisture when compared with controls. Further, our analyses demonstrated that nut interventions are related to an increase in propionate with no effects on acetate and butyrate.

Our synthesis revealed that the effects of nuts on microbiota composition is predominantly selective and modest, with considerably variations between trials. Rather than broad community-wide changes across studies, we observed selective and often nut-specific responses in specific bacterial populations. Although certain genera, including *Clostridium* and *Roseburia*, showed more consistent responses, the direction and magnitude of these changes were not uniform across different nut types. For example, *Roseburia* increased following almond and walnut interventions in 3 trials, yet did not change in response to mixed nut consumption, suggesting the complexity of how different nut varieties may influence specific bacterial populations. Notably, the overall community structure appeared largely resistant to modification by nut consumption, as evidenced by the predominantly null findings for both α-diversity and β-diversity metrics across studies. This stability in diversity measures, combined with the lack of consistent changes in many key bacterial taxa, including Bacillota and Actinomycetota, suggests that the effects of nuts on the gut microbiota may be subtler and more targeted than previously hypothesized. The heterogeneity in observed effects likely stems from multiple factors, including differences in the unique nutrient profiles of different nut varieties, particularly the complexity of fiber content [56] and polyphenol composition among various nut varieties [57]. Evidence suggests that certain polyphenols may either stimulate or inhibit the growth of *Bifidobacterium* species depending on the specific compound [58], supporting the mixed findings for *Bifidobacterium* species.

The null effects of nut treatments on gastrointestinal permeability, as well as the unfavorable effect on fecal moisture, observed in this meta-analysis may be reflective of the relatively healthy study populations recruited in the interventions, the length of the interventions, and variable nut processing methods used. In the study by Burns et al. [26], where constipation was reported on a 5-point Gastrointestinal Symptom Response Scale (with 1 being the lowest possible score indicating no constipation), the mean value was 1.4 following the control intervention compared with 1.2 following the almond intervention. By comparison, scores of 2.6 and 2.4 in people with dyspepsia and irritable bowel syndrome, respectively, have been reported [59]. Regarding the population characteristics, the participants in these trials demonstrated a relatively low initial gastrointestinal symptom burden, and there may be limited opportunity for improvement with nut interventions.

Our meta-analysis revealed a selective effect of nut interventions on SCFAs, specifically demonstrating a significant increase in propionate, whereas acetate and butyrate remained unchanged. A 3-d intervention with walnuts, although not included in the primary meta-analysis due to its brief duration, observed increased concentrations of propionate, which was positively correlated with the abundance of *Phascolarctobacterium* [60], a bacterial genus recognized as an efficient propionate producer [61] and previously linked to propionate production in other in vivo studies [62]. Interestingly, these findings contrast with previous *in vitro* fermentation studies that examined various nut types. Although *in vitro* studies reported increases in all 3 SCFAs, acetate demonstrated the greatest absolute production [56,63]. Substantial heterogeneity was observed among different nut types, with almonds, hazelnuts, and pistachios producing significantly lower SCFA concentrations than cashews—a difference the original authors attributed to variations in glucose concentration [56]. The discrepancies between in vivo and *in vitro* results may be attributed to several methodological and physiological factors. Measuring peripheral SCFAs in plasma, which is distant from the primary site of fermentation in the colon, introduces potential variability as fecal and plasma SCFAs may not correlate [64]. Moreover, other dietary components such as fiber and polyphenols may exert synergistic effects with nut intake, creating a more complex metabolic environment than can be replicated in simplified *in vitro* fermentation experiments.

In our analysis, we observed significant heterogeneity in study designs—including a significant range in the run-in, washout, and intervention durations, disparate DNA extraction and sequencing techniques, and diverse study populations comprising both healthy and at-risk individuals, as well as people who were habitual nut consumers or not. These inconsistencies not only complicate direct comparisons across studies but also introduce substantial variability that may substantially attenuate or artifactually amplify observed effects. In particular, including participants who had habitual nut intake before the intervention might hinder significant effects on the gut microbiota since these people could be accustomed to these food components, different from those who were not regular nut eaters. Further, differences in the DNA extraction technique and the variable region of the 16S rRNA gene that is amplified for sequencing, have been highlighted as sources of variation, which prohibit the direct comparison of results between studies [65,66]. Although these methodological considerations represent potential limitations, they simultaneously present a constructive roadmap for future investigations.

This systematic review and meta-analysis represents an important first step in understanding how nuts influence gut health, although highlighting critical areas for future research. We conducted a comprehensive search strategy enabling a thorough evaluation across various studies. However, the review faced limitations, including a limited number of studies available for each of the nut types and significant heterogeneity in intervention durations and outcomes. Our findings highlight the need for larger well-designed randomized controlled trials examining dose–response effects as well as long-term changes driven by the consumption of the different nut types. Further, standardizing methods for microbiota analysis and study design elements, such as favoring parallel over crossover designs and ensuring consistent intervention durations, is crucial to generate stronger evidence. Additionally, the field would benefit from research clarifying the effects of nut processing and linking microbial changes to health outcomes, as well as understanding individual factors influencing responses. This could help determine whether specific types or combinations of nuts, consumed over a minimum period, are more effective in promoting beneficial microbial changes in specific populations.

In conclusion, this systematic review and meta-analysis revealed that the effects of nut consumption on gut health were discreet, with no effects on gut symptoms and positive effect on 1 SCFA, propionate. Changes in gut microbiota composition were selective and modest, with no clear effects on overall community diversity metrics. Specific bacterial genera showed varying responses. The substantial methodological heterogeneity across studies highlights the need for standardized methodologies to elucidate the specific effects of different nut types on gut microbial composition and function. This will help clarify the mechanisms through which specific nut varieties contribute to gut health and inform dietary recommendations.

## Author contributions

The authors’ responsibilities were as follows—MS: conceived the review, performed the data extraction, conducted the meta-analysis, and wrote the manuscript; JRB: conceived the review, screened the articles identified by the literature search, performed the data extraction, performed risk of bias assessment, and wrote the manuscript; SC: performed the systematic search, performed the data extraction, and read and approved the final manuscript; NS: performed the systematic search update, performed risk of bias assessment, and read and approved the final manuscript; BRC: conceived the review, performed study oversight, screened the articles identified by the literature search, performed the data extraction, and wrote the manuscript; and all authors: read and approved the final version of the manuscript.

## Data availability

The corresponding author will provide data and other materials used in this review upon reasonable request.

## Funding

JRB is supported by a National Health and Medical Research Council Emerging Leadership Fellowship (APP2025943). MS is supported by a National Heart Foundation Postdoctoral Fellowship (106698).

## Conflict of interest

The authors report no conflict of interest.
