# Supplementary material for: Effects of Nut Intake on Gut Microbiome Composition and Gut Function in Adults: A Systematic Review and Meta-analysis
Source: Adv Nutr. 2025 Jun 13;16(7):100465. doi: 10.1016/j.advnut.2025.100465 (PMC12272589; doi:10.1016/j.advnut.2025.100465)
Supplement: Multimedia component 1 [file mmc1.docx]

**The effects of nut intake on gut microbiome composition and gut function in adults: a systematic review and meta-analysis**

Matthew Snelson, Jessica R. Biesiekierski, Susanna Chen, Nessmah Sultan, Barbara R Cardoso

**Supplemental material**

**Supplemental Table 1.** Example of database search strategy used (EMBASE on 21/12/2023, repeated on 07/07/2027)

| **#** | **Search Term** |
| --- | --- |
| 1 | exp nut/ |
| 2 | nut consumption.mp. |
| 3 | (juglans or walnut*).mp. |
| 4 | (anacardium or cashew*).mp. |
| 5 | (prunus dulcis or almond*).mpm |
| 6 | (corylus or hazelnut*).mp. |
| 7 | (pistacia or pistachio*).mp. |
| 8 | (carya or pecan*).mp. |
| 9 | (arachis or peanut*).mp. |
| 10 | (pinus or pine nut*).mp. |
| 11 | (bertholletia or brazil nut*).mp. |
| 12 | or/1-11 |
| 13 | exp microbiome/ or microbiome.mp. |
| 14 | microbiota.mp. |
| 15 | microflora.mp. |
| 16 | microflora/ |
| 17 | bifido*.mp. |
| 18 | Bifidobacterium/ |
| 19 | lactobacill*.mp. |
| 20 | Lactobacillus/ |
| 21 | (faecal or fecal).mp. |
| 22 | bacteri*.mp. |
| 23 | bacterium/ |
| 24 | colon flora/ or bacterial flora/ or intestine flora/ or flora/ |
| 25 | ((colon or bacteri* or intestin*) adj2 flora).mp. |
| 26 | exp dysbiosis/ |
| 27 | dysbiosis.mp. |
| 28 | dysbacteriosis.mp. |
| 29 | 13 or 14 or 16 or 17 or 18 or 19 or 20 or 21 or 22 or 23 or 24 or 25 or 26 or 27 or 28 |
| 30 | "Walnut Consumption Alters the Gastrointestinal Microbiota, Microbially Derived Secondary Bile Acids, and Health Markers in Healthy Adults".mp. |
| 31 | "Almond Consumption and Processing Affects the Composition of the Gastrointestinal Microbiota of Healthy Adult Men and Women".mp. |
| 32 | "Almond Snacking for 8 wk Increases Alpha-Diversity of the Gastrointestinal Microbiome and Decreases Bacteroides fragilis Abundance Compared with an Isocaloric Snack in College Freshmen".mp. |
| 33 | "A Walnut-Enriched Diet Affects Gut Microbiome in Healthy Caucasian Subjects".mp. |
| 34 | "Effects of almond and pistachio consumption on gut microbiota composition in a randomised cross-over human feeding study".mp. |
| 35 | 12 and 29 |
| 36 | 30 and 35 |
| 37 | 31 and 35 |
| 38 | 32 and 35 |
| 39 | 33 and 35 |
| 40 | "Mixed Tree Nuts, Cognition, and Gut Microbiota: A 4-Week, Placebo-Controlled, Randomized Crossover Trial in Healthy Nonelderly Adults".mp. |
| 41 | "Peanuts as a nighttime snack enrich butyrate-producing bacteria compared to an isocaloric lower-fat higher-carbohydrate snack in adults with elevated fasting glucose: A randomized crossover trial".mp. |
| 42 | 35 and 41 |
| 43 | 35 and 40 |
| 44 | "Effects of the Mediterranean Diet or Nut Consumption on Gut Microbiota Composition and Fecal Metabolites and their Relationship with Cardiometabolic Risk Factors".mp. |
| 45 | 35 and 44 |

**Supplemental Table 2.** Methodological details of 16S rRNA sequencing.

| **Study** | **16S region** | **16S Forward Primer** | **16S Reverse Primer** | **Reference Database** | **DNA Extraction Kit** |
| --- | --- | --- | --- | --- | --- |
| Bamburger 2018 (24) | V3-V4 | Not specified | Not specified | Not specified | Not specified, stated modified version of Godon 1997 (PMID: 9212428) |
| Burns 2015 (25) | V1-V3 | 27F | 338R | Not specified | QIAmp Easy DNA Stool Kit, included a bead-beating step |
| Choo 2021 (36) | V4 | 515F | 806R | SILVA (version not specified) | PowerSoil-htp 96-Well Soil DNA Isolation kit |
| Creedon 2022 (50) | V1-V2 | Not specified | Not specified | SILVA - version 132 | DNEasy PowerLyser PowerSoil DNA Isolation Kit (Qiagen) |
| Dhillon 2019 (37) | V4-V5 | 530F | 926R | Greengenes 13_8 | MoBio power soil DNA isolation kit (MoBio Laboratories, Inc) |
| Haskel-Ramsay 2023 (26) | V4 | Not specified | Not specified | Greengenes 13_8 | DNeasy PowerSoil HTP 96 kit (QIAGEN) |
| Herselman 2022 (41) | V3-V4 | 341F | 806R | SILVA (version not specified) | Not specified, states "microbial DNA extraction... was performed by the Australian Genomics Research Facility." |
| Holscher 2018 (28) | V4 | 515F | 806R | Greengenes 13_8 | PowerLyzer PowerSoil DNA Isolation Kit (MoBio Laboratories) |
| Holscher 2018 (29) | V4 | 505F | 806R | Greengenes 13_8 | PowerLyzer PowerSoil DNA Isolation Kit (MoBio Laboratories) |
| Lambert 2020 (30) | V4 | 515F | 806R | SILVA - version 132 | DNeasy PowerSoil HTP 96 DNA Isolation kit (Qiagen) |
| Ren 2020 (47) | V3-V4 | Not specified | Not specified | SILVA (version not specified) | Not specified. |
| Rosas 2023 (48) | Not specified | Not specified | Not specified | Not specified | Not specified: "isolated DNA was sequenced using 16S rRNA gene sequencing at Laragen (Culver City,CA,USA)." |
| Sapp 2020 (33) | V4 | 515F | 806R | SILVA - version 132 | DNeasy PowerSoil kit (Qiagen) |
| Tindall 2020 (34) | V4 | 515F | 806R | SILVA - version 132 | Qiagen Powersoil DNA Isolation kit with lysing performed for 5 min on a Disruptor Genie cell disruptor |
| Ukhanova 2014 (35) | V1-V3 | 27F | 533R | Ribosomal Database Project (version not specified) | Not specified: "Bacterial genomic DNA was isolated from the faecal samples by the bead-beating method." |
| Wang 2021 (45) | V3-V4 | 338F | 806R | SILVA - version 128 | E.Z.N.A. stool DNA Kit (Omega Bio-tek, USA) |
| Yang 2023 (46) | V4 | 515F | 806R | SILVA - version 138 | ZymoBIOMICS DNA Miniprep Kit |
| Souza Silveira 2024 (49) | V3-V4 | 341F | 806R | SILVA - version 138 | Beat-beating and phenol/chloroform extraction protocol |


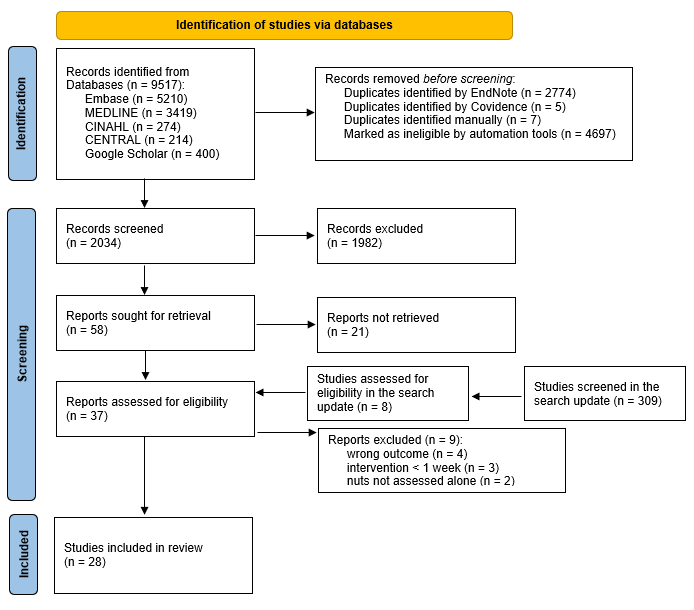


**Supplemental Fig 1.** Flowchart of study selection process.


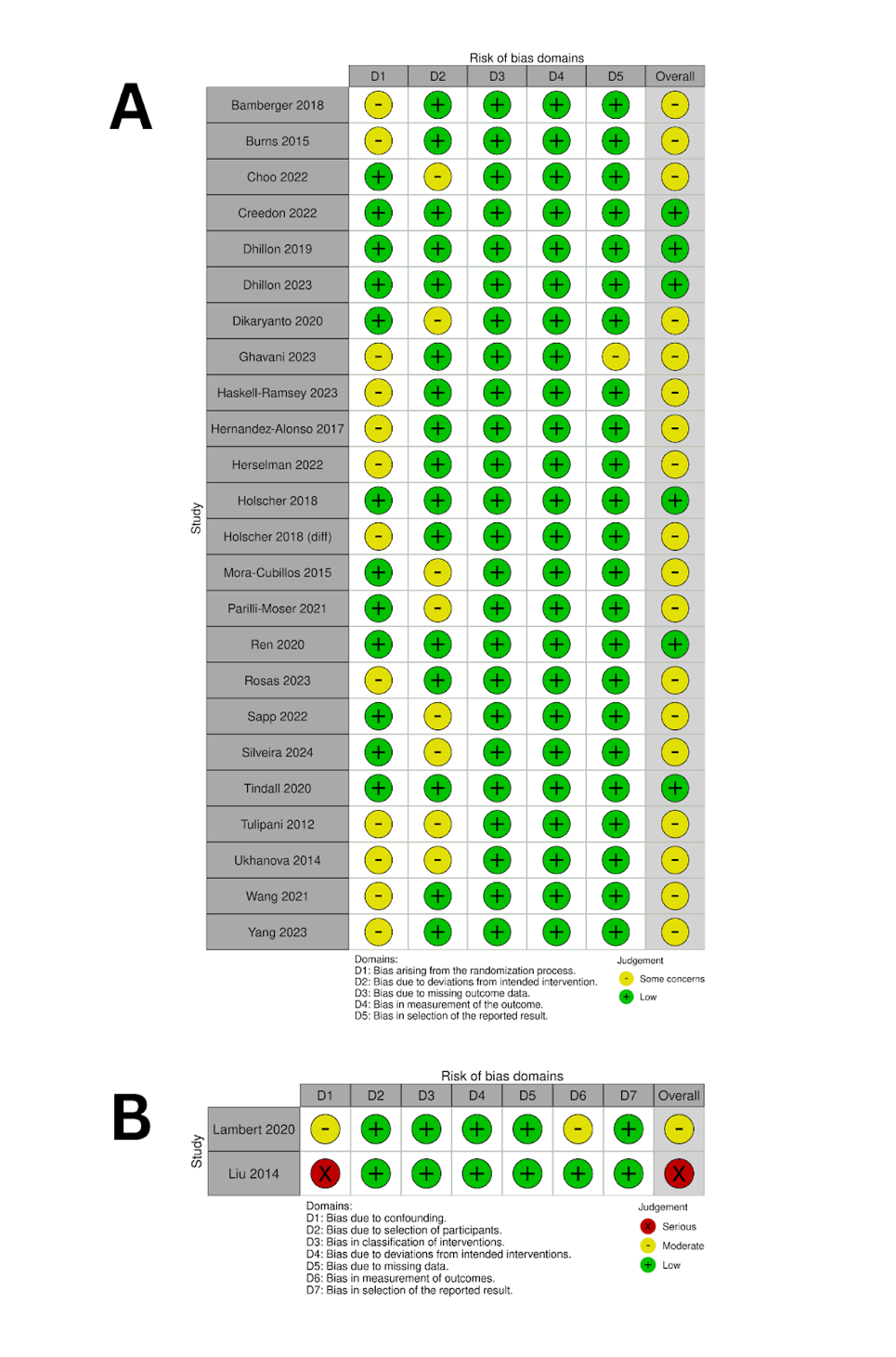

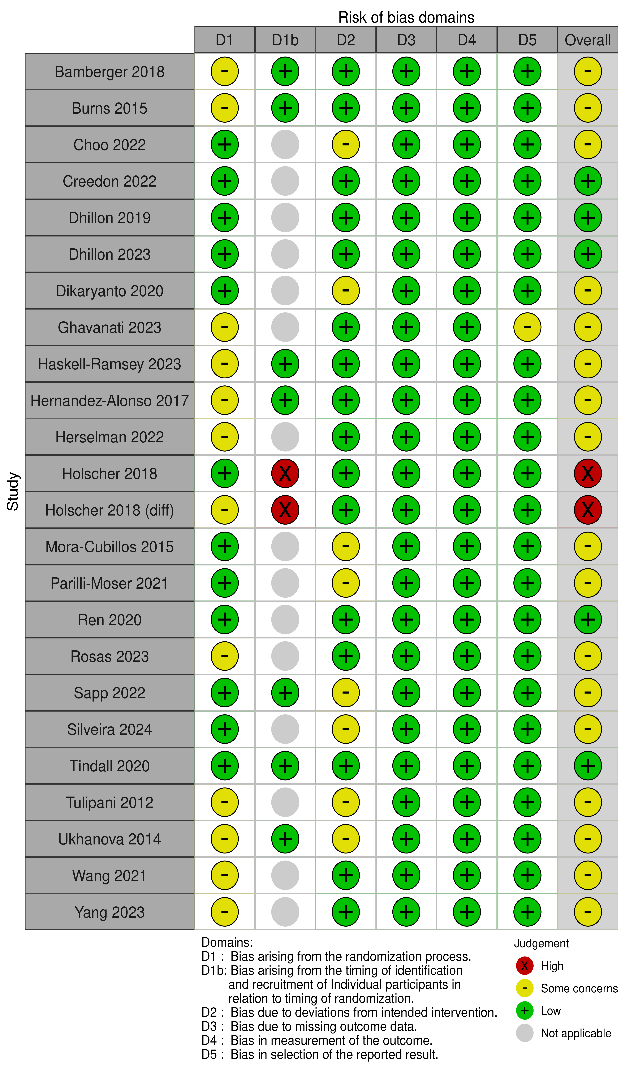


**Supplemental Fig 2.** Risk of bias assessments for randomised controlled trials (A, ROB-2) and non-randomised intervention trials (B, ROBINS-I). For ROB 2 D1, Bias arising from the randomisation process, including baseline differences between intervention groups (D1b); D2, Bias due to deviations from intended intervention; D3, Bias due to missing outcome data; D4, Bias in measurement of the outcome; D5, Bias in selection of reported result. For ROBINS-I: D1, Bias due to confounding; D2, Bias due to selection of participants; D3, Bias in classification of interventions; D4, Bias due to deviations from intended interventions; D5, Bias due to missing data; D6, Bias in measurement of outcomes; D7, Bias in selection of reported result.


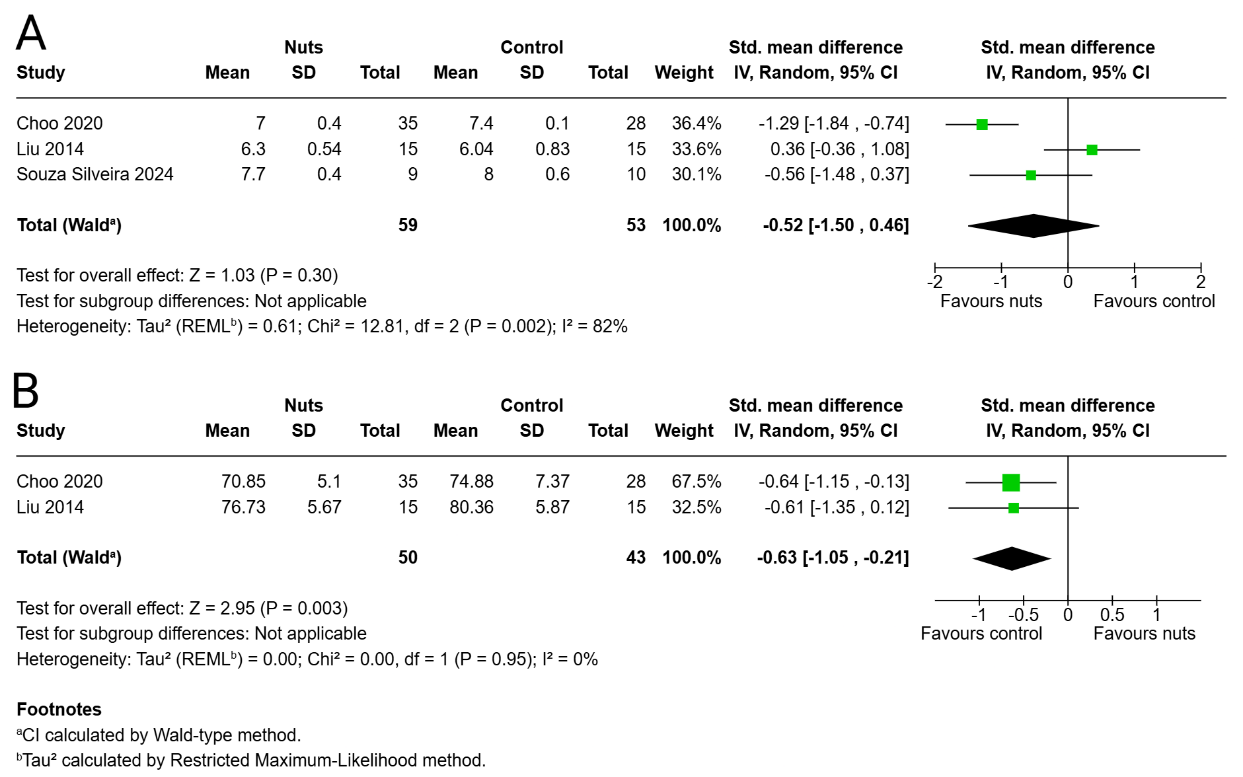


**Supplemental Fig 3.** A sensitivity analysis was conducted excluding the almond skin powder arm of the study by Liu 2014. All other arms in all studies were nuts that were not in butter or skin powder form. Effects of nut consumption on A) faecal pH and B) faecal moisture. Standardised mean difference (95% CI) shown for individual and pooled trials.


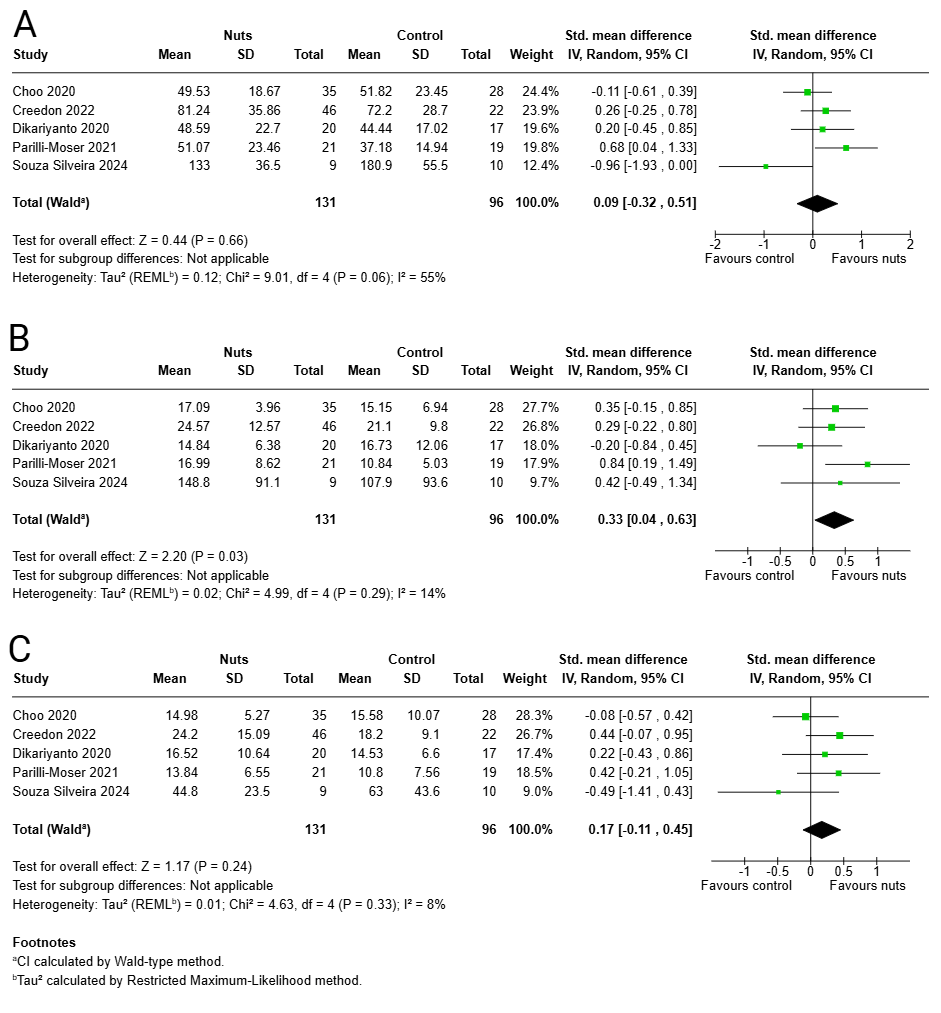


**Supplemental Fig 4.** A sensitivity analysis was conducted excluding the peanut butter arm of the study by Parilli-Moser 2021. All other arms in all studies were nuts that were not in butter or skin powder form. Effects of nut consumption on A) acetate, B) propionate and C) butyrate concentrations. Standardised mean difference (95% CI) shown for individual and pooled trials.
